# Recurrence of Atypical Meningioma: A Case Report

**DOI:** 10.7759/cureus.94686

**Published:** 2025-10-15

**Authors:** Anand Paul, Shilpa Johnson, Shahina M Patel

**Affiliations:** 1 Radiation Oncology, Government Medical College, Thiruvananthapuram, Trivandrum, IND; 2 Internal Medicine, Royal Blackburn Teaching Hospital, Blackburn, GBR; 3 General Internal Medicine, Royal Blackburn Teaching Hospital, Blackburn, GBR

**Keywords:** cns cancer, linac-based sbrt, neuro oncology, neurosurgery, radiation oncology

## Abstract

Atypical meningiomas are classified by the World Health Organization (WHO) as grade II central nervous system tumors, representing an intermediate biological profile between benign (grade I) and anaplastic (grade III) variants. They are defined by elevated mitotic activity, invasive growth patterns, and distinct histopathological features such as hypercellularity, small cell morphology, prominent nucleoli, and areas of necrosis. Clinically, these tumors often manifest with neurological symptoms - headaches, seizures, focal neurological deficits, personality alterations, or visual impairment - depending on their anatomical location. On imaging, atypical meningiomas typically show either homogeneous or heterogeneous contrast enhancement, surrounding edema, and exert mass effect on adjacent brain structures. MRI and MR spectroscopy help in assessing these cases. Surgery tends to be the main treatment, but recurrence is more common than what’s typically seen in grade I meningiomas. Adjuvant radiotherapy is frequently recommended, particularly in cases of subtotal resection or when high-risk features are present. Given their aggressive behavior and propensity for recurrence or malignant progression, comprehensive multidisciplinary care and close surveillance are critical to achieving optimal clinical outcomes. In this case, we present a 57-year-old gentleman diagnosed with peritorcular meningioma.

## Introduction

Meningiomas are the most common primary central nervous system (CNS) tumors, comprising about one-third of all brain and spinal neoplasms. Although generally benign, their CNS location can result in significant morbidity or mortality. In the United States, approximately 37,000 new cases are diagnosed annually, with an incidence rate of 9.7 per 100,000, and a higher prevalence among Black American individuals compared to White American individuals [1]. Incidence increases with age and is notably higher in females, with a female-to-male ratio of 2-3:1, rising to 9:1 in spinal meningiomas [2]. While the etiology is often unknown, prior radiation exposure is a recognized risk factor with a long latency period. Meningiomas are frequently linked to neurofibromatosis (NF2)-related schwannomatosis, and somatic NF2 mutations may contribute to sporadic cases [3].

Atypical meningiomas (WHO Grade II) differ from benign meningiomas (Grade I) by their increased cellularity, elevated mitotic index, architectural disarray, nuclear atypia, and potential for brain invasion or necrosis - features absent in benign forms. While Grade I tumors are slow-growing with low recurrence and a favorable prognosis, Grade II lesions exhibit more aggressive behavior and a higher likelihood of recurrence even after complete resection.

This case highlights the clinical significance of atypical meningioma's aggressive nature and high recurrence risk in a 57-year-old male who underwent surgical resection followed by adjuvant radiotherapy, underscoring the challenges posed by limited therapeutic options in subsequent management.

## Case presentation

A 57-year-old man presented with a three-week history of headache and sudden-onset behavioral disturbances, characterized by irrelevant speech, along with gait instability, reduced walking speed, and slurred speech. The headache was progressive in nature and was usually experienced in the morning, not associated with any visual disturbances, vertigo, vomiting, seizures, or other neurological symptoms. There were no known comorbidities; however, he had a history of chronic smoking and occasional alcohol consumption. Family history was non-contributory. Systemic examination was normal, and neurological assessment revealed ataxia and dysarthria, with preserved cranial nerve function. Laboratory investigations were within normal limits (Table 1).

**Table 1:** Baseline investigation at the time of presentation.

Investigations	Values	Normal range
Hemoglobin	15.6 g/dL	13.5-17.5 g/dL
Total count	5100 cells/µL	4000-10000 cells/µL
Platelet	3.3 lakh cells/µL	1.5-4 lakh cells/µL
Urea	36 mg/dL	8-24 mg/dL
Creatinine	0.9 mg/dL	0.6-1.3 mg/dL
Sodium	132 mEq/L	135-145 mEq/L
Potassium	4.3 mmol/L	3.5-5.2 mmol/L
Bilirubin total	1.4 mg/dL	0.3-1.2 mg/dL
Bilirubin direct	0.3 mg/dL	0.3-1.2 mg/dL
Serum glutamate pyruvate transaminase	29 U/L	7-56 U/L
Serum glutamic oxaloacetic transaminase	21 U/L	5-40 U/L

CT brain in June 2024 showed no evidence of intracranial hemorrhage. MRI in July 2024 revealed a well-defined extra-axial lesion in the infratentorial posterior fossa that appeared T2-weighted (T2) and fluid-attenuated inversion recovery (FLAIR) hyperintense and T1-weighted (T1) isointense, with homogeneous post-contrast enhancement, mild diffusion restriction, and focal blooming on susceptibility-weighted imaging (SWI) in the right latero-inferior aspect. The lesion extended inferiorly to the foramen magnum with minimal cervical canal involvement, and superiorly abutted the dura with nodular thickening along the tentorium cerebelli, showing supratentorial continuity. It caused anterior compression of the cerebellar hemispheres and vermis, leading to complete effacement of the fourth ventricle and upstream hydrocephalus with transependymal edema. MR spectroscopy showed a significant choline peak without a definite alanine peak. Measuring approximately 3.3 × 6.6 × 7.5 cm, the lesion also invaded the straight sinus posteriorly along the right lateral wall, with a central non-enhancing filling defect suggestive of thrombus.

**Figure 1:**
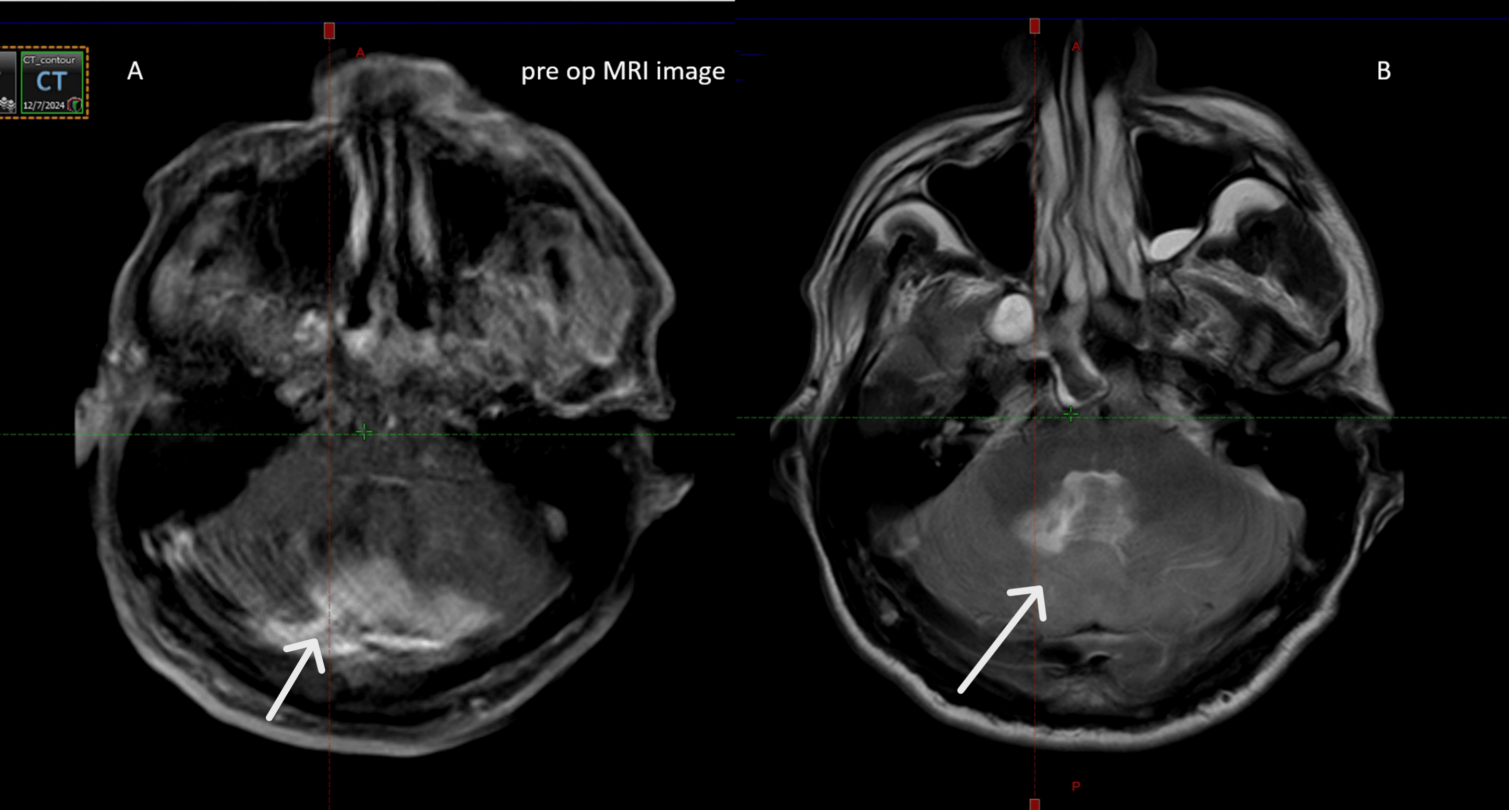
(A, B) Preoperative MRI (axial cuts) showing a hyperdense lesion suggestive of an atypical meningioma (arrows).

MRI showing a well-defined extra-axial lesion in the infratentorial posterior fossa, T2/FLAIR hyperintense and T1 isointense (Figure 1). The lesion extended inferiorly to the foramen magnum with minimal cervical canal involvement. It caused anterior compression of the cerebellar hemispheres and vermis, measuring approximately 3.3 × 6.6 × 7.5 cm.

Metastatic workup was negative. The patient was diagnosed with atypical infratentorial peritrocular meningioma (WHO Grade 2) and underwent midline suboccipital craniotomy with Simpson’s grade 2 excision, duroplasty, and mesh cranioplasty on 27/08/2024. Intraoperatively, the tumor was attached to the inferior leaflet of the tentorium, extending to the incisura and foramen magnum, displacing the cerebellar vermis and hemispheres anteriorly, with dural infiltration. It appeared greyish, friable, firm, moderately vascular, and adherent to the dura and tentorium, and was excised piecemeal. Postoperative CT on 29/08/2024 showed bilateral cerebellar heterogeneity and occipital calvarial deficit consistent with surgical changes. Histopathology revealed a lobulated and diffuse sheet-like neoplasm with occasional whorling, pleomorphic vesicular nuclei, prominent nucleoli, increased cellularity, and 7-8 mitoses per 10 high-power field. Immunohistochemistry showed diffuse vimentin positivity, patchy anti-endomysial antibody (EMA) positivity, and negativity for synaptophysin, cytokeratin, and glial fibrillary acidic protein (GFAP); MIB-1 index was 10-15%. Findings confirmed atypical meningioma (WHO Grade 2).

The patient was referred from neurosurgery for adjuvant radiotherapy. Postoperative MRI brain (November 2024) showed expected surgical changes in the posterior fossa with mesh in situ and mild atrophy of the parasagittal cerebellar hemispheres with T2/FLAIR hyperintensity. No residual enhancing lesion was identified at the surgical site. A ventricular shunt was noted in the right cerebral hemisphere, with mild pericatheter FLAIR hyperintensity. Scattered T2/FLAIR hyperintensities were seen in the bilateral deep white matter without diffusion restriction or blooming. The rest of the brain parenchyma, including grey-white matter differentiation, basal ganglia, thalami, midbrain, and pons, appeared normal. Magnetic resonance venography (MRV) showed non-visualization of the right transverse sinus, likely due to shunt-related artifact. No hydrocephalus or residual disease was evident.

Following a multidisciplinary team (MDT) discussion, the patient received adjuvant radiotherapy of 54 Gray in 30 fractions over five weeks using 3D conformal radiotherapy (3DCRT) after immobilization with a thermoplastic Orfit shell (Orfit Industries NV, Wijnegem, Belgium) on a Linear Accelerator (LINAC IX; Varian Medical Systems, Palo Alto, California, USA). Target volume delineation incorporated postoperative changes and preoperative MRI-defined disease within the clinical target volume (CTV). A planning target volume (PTV) margin of 3 mm was applied around the CTV.

**Figure 2:**
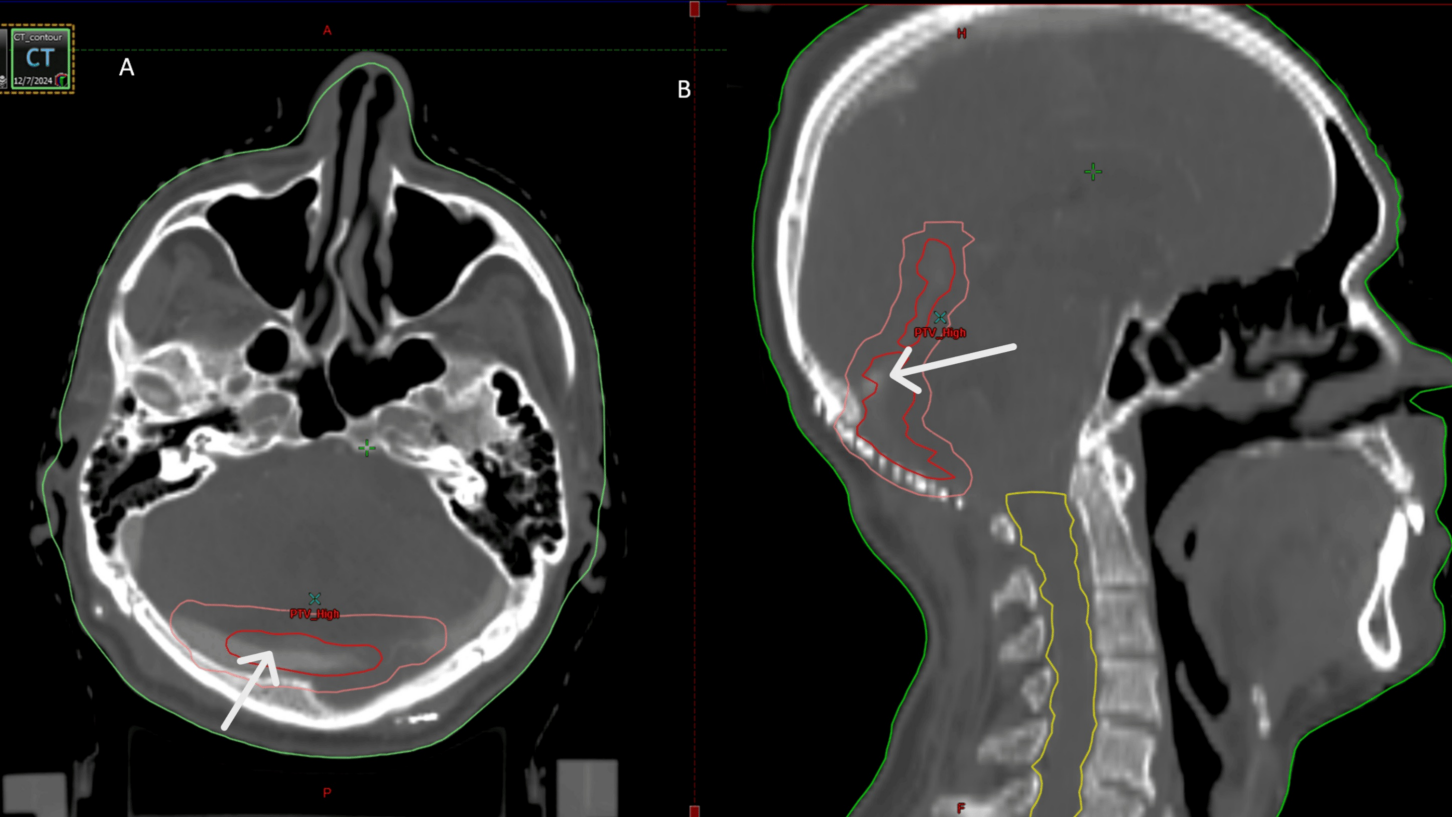
Contouring images: (A) Axial view showing the clinical target volume (CTV) in red and the planning target volume (PTV) in pink. (B) Sagittal view showing the CTV in red, the PTV in pink, and the spinal canal contoured in yellow.

The patient completed treatment in February 2025 with good tolerance and a normal postoperative CNS examination. After MDT discussions, there was no indication of adjuvant therapy. Initial follow-up was uneventful. A three-month post-radiotherapy CT brain revealed heterogeneous hypodensity in the bilateral cerebellar hemispheres near the calvarial defect with fourth ventricle dilatation, suggestive of encephalomalacia. Neurosurgical advice was to continue observation (Figure 2).

**Figure 3:**
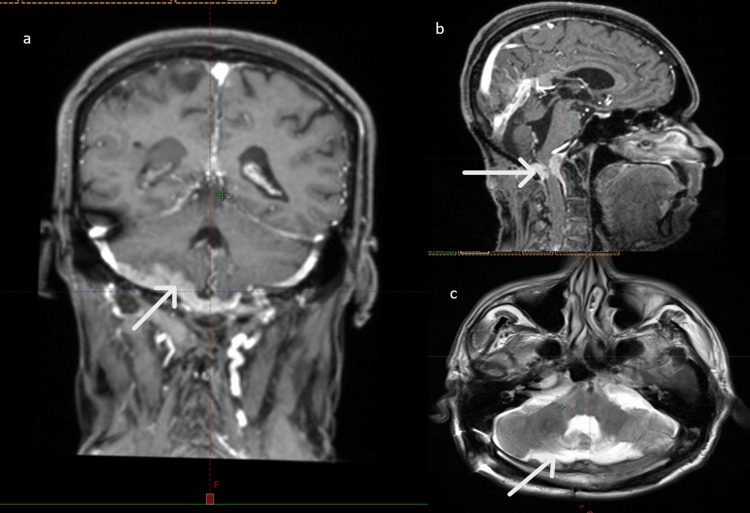
A relatively well-defined extra-axial lesion in the posterior cranial fossa on the right anterior aspect, appearing heterogeneously isointense on T2/FLAIR and hypointense on T1. The lesion shows heterogeneous post-contrast enhancement with extension into the superior aspect of the spinal canal, causing spinal cord compression. A skip lesion is also noted inferiorly within the spinal cord. The lesion appears as hyperdense areas in sagittal (B), coronal (A), and axial (C) images. FLAIR: fluid-attenuated inversion recovery

Due to new behavioral changes and ataxia, MRI showed a recurrent/residual atypical meningioma in the right anterior-posterior cranial fossa with heterogeneous enhancement, extension into the spinal canal causing cord compression, skip lesions, and tumor thrombus in the right sigmoid sinus. Additional enhancing lesions were noted in the parasagittal region and around the straight sinus, suggesting residual disease. Chronic infarcts were seen in the bilateral frontoparietal deep white matter, with no evidence of obstructive hydrocephalus. Histopathology correlation was advised (Figure 3).

The case was reviewed with the neurosurgical team to evaluate the feasibility of decompression, as additional radiotherapy was no longer an option. The patient was referred to neurosurgery and subsequently underwent decompressive surgery. Plans were then initiated for palliative care.

Table 2 presents the summary of CNS evaluation at various time periods.

**Table 2:** Summary of central nervous system evaluation. RT: radiotherapy

	Before surgery	After RT	Post-RT six months
Symptoms	Headache, behavioral changes	Mild swaying	Headache, behavioral changes, neck pain
Higher function	Conscious oriented	Conscious oriented	Short-term loss of memory
Glasgow Coma Scale (GCS)	E4V5M6	E4V5M6	E4V5M6
Mini-Mental State Examination (MMSE)	29/30	29/30	29/30
Bulk of the muscle	Normal	Normal	Normal
Tone of the muscle	Normal	Normal	Normal
Power of muscles in all limbs	5/5	5/5	5/5
Cerebellar function	Ataxia present, dysarthria present	Normal	Ataxia, dysarthria
Cranial nerve examination	Normal	Normal	Mild dysphagia

Table 3 presents the Karnofsky Performance Status (KPS) scores at various time intervals. The full KPS scoring system is provided in Appendix A.

**Table 3:** Karnofsky Performance Status (KPS) score at various time intervals.

	KPS score
Before surgery	70
After surgery	60
Post-radiotherapy	90
Post-radiotherapy six months	50

## Discussion

The WHO 2016 classification designates atypical meningioma (AM) as Grade II, between benign (Grade I) and anaplastic (Grade III) forms. Grades II and III are more frequent in males, with a median diagnosis age of 57 years. MRI is the primary diagnostic tool, with features such as heterogeneous signal intensity, irregular lobulated margins, peritumoral edema, and rapid growth suggesting atypical pathology [4]. However, imaging alone lacks specificity, as benign tumors may present similarly. A reduced apparent diffusion coefficient (ADC), often seen in AM, correlates inversely with the Ki-67 index due to increased cellularity and mitotic activity [5].

Magnetic resonance spectroscopy may reveal elevated choline (Cho) and choline-to-N-acetylaspartate (Cho/NAA) ratios in atypical meningiomas (AMs), aiding differentiation from benign variants [6]. Histopathological confirmation is essential, with brain invasion now a key diagnostic criterion - defined as tumor infiltration into parenchyma without pial separation. AMs typically exhibit infiltrative growth, destroying adjacent arachnoid and pia mater, and features like brain invasion, high mitotic rate, and sheet-like cell arrangement are linked to higher recurrence and shorter disease-free survival. Diagnostic consistency varies, with spontaneous necrosis showing the highest inter-observer agreement. Immunocytochemistry supports diagnosis, with epithelial membrane antigen (EMA) and vimentin as key markers - EMA tends to decrease in high-grade tumors, while vimentin shows diffuse positivity [7]. Negative GFAP and S-100 help exclude neuroepithelial tumors. Most AMs show Ki-67 indices above 5%, and elevated levels are associated with recurrence. Among histological features, Ki-67 and mitotic count are the most reliable predictors of recurrence [8].

While histology remains the diagnostic standard, molecular profiling is increasingly valuable. NF2 mutations and chromosome 22 deletions are common in AMs, and methylation patterns may offer better behavioral stratification. Genes like NF2, SMO, TERT, and TRAF7 are being studied for future classification and targeted therapies [9].

Meningiomas are highly vascular tumors, with vascular endothelial growth factor-A (VEGF-A)-driven angiogenesis playing a central role in their progression, especially in recurrent malignant forms [10]. Bevacizumab, an anti-VEGF monoclonal antibody, has demonstrated disease stabilization in phase II trials [11]. Immunotherapy has shown mixed results - nivolumab yielded limited benefit, while pembrolizumab achieved progression-free survival in high-grade cases, sparking interest in combining anti-VEGF and anti-PD-1 therapies [12,13].

Surgical resection remains the primary treatment, with Simpson grades I-II resections offering superior outcomes. Challenges arise when tumors involve critical neurovascular structures or venous sinuses. Complete removal, including affected dura and bone, significantly improves survival and progression-free intervals [14,15]. Adjuvant radiotherapy remains controversial; recurrence rates without it may reach 48% at 10 years [16,17]. Radiotherapy is generally advised for incomplete resections or aggressive histology, with trials like EORTC 22042 [18], RTOG 0539 [19], and ROAM/EORTC-1308 [20] evaluating its role in grade II/III meningiomas.

In our case, as the patient had already received radiotherapy, re-irradiation was not feasible due to the site. Therefore, he was referred to neurosurgery for decompressive intervention. Due to financial constraints and a lack of phase 3 trials, immunotherapy was not pursued.

## Conclusions

This case illustrates the uncommon infratentorial peritorcular presentation of atypical meningioma, with recurrence marked by venous sinus infiltration and drop metastasis. Given its aggressive behavior, surveillance involves early postoperative MRI, followed by imaging every three to six months for two years and annually thereafter, with intensified follow-up and adjuvant therapy for high-risk or incompletely resected cases. Atypical meningioma is biologically heterogeneous and locally invasive; while imaging aids initial assessment, definitive diagnosis depends on histopathology and molecular profiling. Complete surgical excision remains the primary strategy to minimize recurrence. In relapsed cases, standardized treatment is lacking; although immunotherapy trials are underway, their effectiveness is yet to be established. When re-irradiation is not an option, a tailored multimodal approach should be considered.

## Appendices

Appendix A

**Table 4:** Karnofsky Performance Status (KPS) score table

KPS Score Table	
Score	Description
100	Normal; no complaints; no evidence of disease
90	Able to carry on normal activity; minor symptoms
80	Normal activity with effort; some symptoms
70	Cares for self; unable to carry on normal activity
60	Requires occasional assistance but can care for self
50	Requires considerable assistance and frequent care
40	Disabled; requires special care and assistance
30	Severely disabled; hospitalization indicated
20	Very sick; active supportive treatment needed
10	Moribund; fatal processes progressing rapidly
0	Dead
